# Perceptions and Practice of Early Diagnosis of Sickle Cell Disease by Parents and Physicians in a Southwestern State of Nigeria

**DOI:** 10.1155/2020/4801087

**Published:** 2020-05-31

**Authors:** Oladele Simeon Olatunya, Adefunke Olarinre Babatola, Ezra Olatunde Ogundare, Babatunde Ajayi Olofinbiyi, Olubunmi Adeola Lawal, Jacob Olumuyiwa Awoleke, Olusola Peter Aduloju, Alaba Olanrewaju Daramola, Eyitayo Ebenezer Emmanuel, Oyebanji Anthony Olajuyin, Akinwumi Kolawole Komolafe, Abiola Olukayode Olaleye

**Affiliations:** ^1^Department of Paediatrics, Ekiti State University, Ado Ekiti, Ekiti State, Nigeria; ^2^Department of Paediatrics, Ekiti State University Teaching Hospital, Ado Ekiti, Ekiti State, Nigeria; ^3^Department of Obstetrics & Gynaecology, Ekiti State University, Ado Ekiti, Ekiti State, Nigeria; ^4^Department of Paediatrics, Federal Teaching Hospital, Ido Ekiti, Ekiti State, Nigeria; ^5^Department of Hematology, Ekiti State University, Ado Ekiti, Ekiti State, Nigeria; ^6^Department of Community Medicine, Ekiti State University, Ado Ekiti, Ekiti State, Nigeria; ^7^Department of Ear Nose & Throat, Ekiti State University, Ado Ekiti, Ekiti State, Nigeria; ^8^Department of Community Medicine, Obafemi Awolowo University, Ile-Ife, Osun State, Nigeria

## Abstract

**Background:**

Early sickle cell disease (SCD) diagnosis has shown promise in combating SCD in many countries. The aim of this study was to assess the practice and perception of early SCD diagnosis among a group of parents and physicians in Nigeria. *Patients and Methods*. This was a cross-sectional descriptive study conducted to assess the opinions and practice of early diagnosis of SCD among 135 physicians caring for SCD patients and 164 mothers of children with SCD in a southwestern state of Nigeria.

**Results:**

Most physicians 132 (97.8%) were aware of prenatal SCD diagnosis, but only 51 (37.8%) would recommend it. Most physicians 129 (95.6%) routinely recommend premarital SCD genetic counseling and testing, and 89 (65.1%) were aware of the national government newborn screening program but lesser proportion 75 (55.6%) were willing to recommend it. Amongst the mothers, 154 (94%) and 158 (96%) had encountered genetic counseling for SCD and were willing to offer newborn screening to their children, respectively. On the contrary, fewer mothers 42 (25%) were aware of prenatal SCD diagnosis, 28 (17%) were willing to partake in it, and 44 (26%) were undecided. There were discrepancies in the willingness by physicians to practice early SCD diagnosis and its uptake by mothers (*p* < 0.0001). The commonest reason given by both the physicians and mothers for not practicing SCD prenatal diagnosis was the high cost of the procedure.

**Conclusion:**

The perceptions and practice of early SCD diagnosis was suboptimal in the study locality. Scaling up awareness and universal coverage are required.

## 1. Introduction

Sickle cell disease (SCD) is a common genetic disorder of man that is caused by a mutation in the *β*-globin gene [1, 2]. Individuals homozygous for the sickle hemoglobin gene (*HbS*) have the most severe form of the disease known as sickle cell anemia [1, 2]. Patients with SCD exhibit clinical variability both within individuals and across groups of patients, and the prevalence of the disease is high in sub-Saharan Africa (SSA) [1–3]. SCD is a disorder of public health concern especially in sub-Saharan Africa (SSA) where approximately 75% of the global 312,000 births of affected newborns occurred in 2010 [3], and the majority between 50 and 80% of affected children in this part of the world die before adulthood [1–3].

According to the Global Burden of Diseases (GBD), in 2010, sickle cell disorders accounted for 0.42 deaths per 100,000; 28.69 years of life lost (YLLs) per 100,000 and 53.21 years lived with disability (YLDs) per 100,000, adding up to 81.9 disability-adjusted life years (DALYs) per 100,000 [4]. Nigeria has the highest burden of the disease where between 1% and 2% of newborns are born annually with the medical condition. It is also an important cause of childhood morbidity and mortality in the country [5, 6].

The World Health Organization (WHO) estimates that 70% of SCD deaths in Africa are preventable with simple, cost-effective interventions such as early identification of SCD patients by newborn screening (NBS) and subsequent provision of comprehensive care [7, 8]. Adoption of these practices have led to the achievement of significant reduction in SCD mortality with more than 90% of affected children now surviving through adulthood in some parts of the world [9]. On the contrary, most countries in SSA where the huge burden of the disease reside still lag behind in the implementation of these cost-proven measures that have worked in the developed countries. Hence, the continued high SCD morbidity and mortality rates in Africa [5–7, 10].

In recognition of the high burden of sickle cell disease in Nigeria, the Federal Government of Nigeria has recently taken some practical steps to lessen this burden. These include the initiation of a staggered regional-based newborn screening (NBS) programme in 2012 by starting a stepwise NBS programme beginning with six centres (one centre located in each of the six geopolitical zones of the country) and this was followed up with the launch of the national guideline for SCD management and control in 2014 [11]. However, there has not been attempt to evaluate how these measures have impacted SCD in the country since their introduction. Given that physicians and parents are major stakeholders in the effective implementation of any government health policy, it is important that their views and practice of the new policies are assessed. The aim of this study was to assess the awareness of the national sickle cell disease NBS programme as well as the practice and perception about early diagnosis of SCD among parents and physicians in a southwestern state of Nigeria.

## 2. Patients and Methods

### 2.1. Study Design and Setting

This was a cross-sectional descriptive study conducted among physicians and mothers of children with SCD in Ekiti State between September and December 2018. The study settings were public and private hospitals located within the state. These two groups of hospitals were selected because majority of the populace usually patronize them for their health care delivery.

Ekiti State is located between longitudes 4° 45° and 5° 45° east of the Greenwich Meridian and latitudes 7° 15° and 8° 15° north of the equator in Southwest Nigeria. Agriculture is the main occupation of the citizens, providing income and employment for more than 75% of the population [12]. It has 16 Local Government Areas and a population of 3,270,798 according to the Nigerian National Bureau of Statistics [12].

There are many public and private health facilities in the state. These health facilities provide different levels of care to patients with SCD based on facilities available in them. The Ekiti State University Teaching Hospital (EKSUTH) is located in the state capital and is the core centre for SCD care in the state. It provides holistic care for SCD patients in the state and other adjoining neighborhood states with the collaboration of various specialists in the hospital. It also serves as the referral centre to other health facilities in the state. The EKSUTH has a SCD support club which was started in 2014 where repeated health talks and information on SCD are given to caregivers (parents/guardians) through group discussions, audio-visual aids, information booklets, books, etc. At the EKSUTH, the determinations of Hb genotypes of pregnant mothers as well as those for premarital testing are done by using cellulose acetate Hb electrophoresis in an alkaline buffer.

### 2.2. Study Participants and Sampling

This study was conducted among physicians caring for SCD and mothers of children with SCD. The participants were purposively selected to ascertain their awareness of the current efforts of the Nigeria government at combating SCD. Specifically, their awareness and opinions on NBS using the special newborn program of high-performance liquid chromatography (HPLC) provided by the Federal Government of Nigeria and prenatal diagnosis through chorionic villus sampling and amniocentesis were assessed. The physicians were drawn from health institutions across the state. They were those involved in the direct care of patients with SCD and practicing in the state. The physicians comprised resident doctors, medical officers, and specialist consultants from various departments and hospitals who were involved in the care of patients with SCD in the state. The mothers were those of children with SCD who were regular at both the sickle cell clinics and the Pediatric Hematology Unit of the Ekiti State University Teaching Hospital, Ado Ekiti. This group of mothers was purposively chosen because of their participation in the EKSUTH SCD support club activities where some health education on SCD are usually given and as such were presumed to have some knowledge of SCD.

### 2.3. Data Collection and Study Instrument

Information was obtained from the participants through questionnaires. The questionnaire has sections on biodemographic data, knowledge, and awareness of government programmes on NBS, national SCD management guidelines, and perception on premarital counseling and prenatal diagnosis of SCD. The questionnaires were pretested among a few physicians and parents from a neighbouring hospital, and their responses were used to improve the study instrument before being used for the main study. Those who participated in the pretesting of the questionnaire, as well as those who declined to participate, were exempted from the study. The opinions of the two groups of participants on awareness of SCD treatment guidelines and their practice/beliefs of early SCD diagnosis were assessed.

The contacts of the physicians were obtained from the secretariats of their relevant professional bodies, and the pretested questionnaires were delivered to the physicians manually with the help of trained research assistants who also retrieved the same from the physicians. Only physicians in charge of SCD care at their various hospitals were urged to fill the questionnaire. The questionnaire was self-administered by the physicians. The questionnaire was translated into local languages for the mothers who did not understand English language. In order to ensure correct translation of the questionnaire to the mothers, three junior doctors who understood the local languages in addition to the official English language were trained to administer the questionnaires to mothers of children with SCD attending the EKSUTH. The principal investigator checked at random the completed questionnaire to ensure correctness and reliability of recorded data.

### 2.4. Ethical Consideration

The study protocol was approved by the Ethics and Research Committee of the Ekiti State University Teaching Hospital, Ado Ekiti.

The participants were informed about the study, and the purpose of the study was explained to them in clear and plain language. The consent of each participant was sought through an information/consent note attached to the questionnaire.

Participation was voluntary, and only physicians and mothers who consented to participate were recruited. The decision of the mothers to participate or not did not interrupt or affect the care given to their wards, as all patients received the same standard treatments without discrimination.

### 2.5. Data Analysis

The opinions of the two groups of participants on their practice/beliefs of early SCD diagnosis were assessed. The Statistical Package for Social Sciences (SPSS) version 20 (IBM, USA) was used to analyze the data using descriptive and comparative statistics. The level of significance was set at *p* < 0.05.

## 3. Results

A total of 162 physicians were contacted, out of which 135 responded, giving a response rate of 83.3%. Also, 164 mothers participated in the study. Of the 135 physicians that responded, 69 (51.1%) were males. The majority of the physicians 120 (88.9%) were in government employment. Seventy three (54.1%) physicians had less than a decade of practice experience. One hundred and three (76.3%) of them experienced premarital SCD genetic counseling and testing before getting married. Nearly all, 129 (95.6%), physicians routinely recommend premarital SCD genetic counseling and testing to their eligible clients (Table 1).

Almost two-thirds of the physicians, 89 (65.1%), was aware of the national government newborn screening project, but lesser proportion 75 (55.6%) were willing to recommend it. The major reason proffered for their unwillingness to recommend NBS was lack of access to it, 14 (10.4%). Only 96 (71.1%) physicians were aware of the National Guideline for the Control and Management of SCD in Nigeria. However, almost all, 132 (97.8%), were aware of prenatal SCD diagnosis. Only 51 (37.8%) would wish to recommend prenatal diagnosis of SCD if indicated, while others were opposed to it (Table 1).

As shown in Table 2, most of the mothers were literate and married. Also, most of them 159 (97%) had only one child with SCD. However, one mother has three children with the condition. Similarly, 154 (94%) mothers had been exposed to genetic counseling for SCD, and 158 (96%) were willing to accept NBS for their future children. On the contrary, only 42 (25%) of the mothers were aware of prenatal diagnosis, with a lesser proportion, 28 (17%), willing to partake in it in the future. Forty four mothers (26%) were undecided.

As shown in Table 3, physicians were more aware about the guideline for SCD treatment and newborn screening when compared to the mothers (*p* < 0.0001). Also, they were more aware about prenatal diagnosis and more willing to recommend it compared to the mothers willingness to partake in it (*p* < 0.0001). On the contrary, the physicians were less willing to recommend newborn screening while more mothers were willing to partake in it (*p* < 0.0001) (Table 3). All the 14 (100%) physicians who were not willing to recommend NBS gave unavailability/inaccessibility as the reason for their decision while a paltry 4 (28.6%) hinged their decision on economic reasons.

The commonest reason given by both the physicians and mothers for not practicing SCD prenatal diagnosis was the high cost of the procedure. Other reasons given by the physicians for not practicing prenatal SCD diagnosis differed from those of the mothers. More mothers compared to physicians (33.7% vs 13%) *p*=0.0014, gave fear of injury to the baby or mother as reasons for nonwilling to practice prenatal SCD. On the contrary, more physicians compared to mothers (15.5% vs 2.2%) *p*=0.002, gave previous negative experience as reasons for nonwilling to practice/recommend prenatal SCD. However, 25% of doctors not willing to offer prenatal diagnosis gave ethical issues such as counseling for, and performing, termination of pregnancy (TOP) as treatment options if the mother requests as the reason for their decisions (Table 4).

## 4. Discussion

The high burden of SCD in sub-Saharan Africa and its attendant high morbidity and mortality raise the need for more investment in ways to reduce its burden in these countries. Nothing drives home this point than the observation by the World Health Organization (WHO) which estimates that 70% of SCD deaths in Africa are preventable with simple, cost-effective interventions such as early identification of SCD patients by newborn screening (NBS) and subsequent provision of comprehensive care [7, 8].

Drawing from the above, the high level of willingness of participants to partake in newborn screening is commendable and may be a step towards combating the huge scourge of SCD in the country as has been found in many countries where NBS is being implemented [13–20]. The newborn screening program allows for early diagnosis, parental education, and comprehensive care, which results in a marked positive impact on mortality and morbidity throughout infancy, childhood, and adulthood in many countries where it is being practiced [14–20]. The United States of America (USA) was the first country to implement a universal newborn screening programme for SCD in 1975 following the enactment of the Sickle cell Anemia Act of 1972, and by 2006, all the 50 states were already practicing it [13]. In France, a targeted programme for at-risk groups is being implemented [14]. Also, the United Kingdom (UK) commenced universal newborn screening in 2006 through the National Health Service (NHS). However, its impact on the SCD prevalence is still debatable [15, 16].

Contrary to the observation in the UK, [15, 16] vis-a-vis the impact of NBS, recent data from the New York newborn screening program highlighted that the mortality rate was significantly reduced among children of foreign-born mothers, majority of whom were Africans [17]. In addition, the Dallas newborn cohort of children with SCD in USA between 2000 and 2007 recorded no single death in childhood due to prompt diagnosis and subsequent comprehensive care made possible through newborn screening [18]. Furthermore, newborn screenings have also led to huge decline in the prevalence and morbidity/mortality from SCD between 2004 and 2009 in Saudi Arabia [19]. In Bahrain, another Middle Eastern nation, NBS led to a decline in SCD prevalence from 2.1% in 1985 to 0.4% in 2010 [20]. These observations [13–20] attest to the positive impacts of NBS if properly implemented.

However, as found in this study and akin to what obtains in many African countries [7, 21–23], there is no holistic or universal NBS, except for pockets of regional, local, or phased screening in countries like Tanzania [7], Ghana, [21], Benin [22], and Democratic Republic of Congo (DRC) [23]. Although the launch of the scheme in Nigeria in 2012 is apt and laudable, it is yet to attain universality. Efforts should be targeted at scaling up awareness of the scheme so that the intended population targets could be reached in order to avert the accessibility problems raised by some physicians in this study. This will help to assuage the previous observation that children with SCD still have a high risk of dying from complications of SCD without being diagnosed at all due to the lack of universal newborn screening in most low-income countries, particularly in Africa [24].

Earlier studies have revealed that genetic counseling for SCD is mainly practiced in Nigeria in a manner similar to the Cypriot model, where the practice was mainly domicile with religious bodies [25–27]. Nevertheless, the high willingness and uptake of genetic counseling in this study is commendable and could be a step in the right direction towards mitigating the burden of SCD in the locality and by extension, in Nigeria as opined by previous authors [25–27]. Premarital genetic counseling allows couples to know their SCD risk and plan the health needs of their family. It also gives them the opportunities available to them vis-a-vis choices on their family size, prenatal, or NBS and provision of comprehensive care for their would-be affected wards. Hence, we recommend that stakeholders in the health sector should not hesitate to partner with the religious bodies to further scale-up SCD genetic counseling in the study locality given the current dominance of religious bodies in the practice in the country.

Compared with the physicians interviewed, the lack of awareness of the national guideline for SCD management and control by the mothers is understandable, given that most of them are probably medically lay persons. Nonetheless, less scientific/medically worded education materials could be made available by the government to the locals in the study area in order to complement the efforts of stakeholders at making lay persons like parents more knowledgeable in SCD care as suggested earlier [11]. This line of thought was also buttressed by Reagan et al. [28] who found that health education of stakeholders and the implementation of treatment guidelines to manage some SCD acute events led to a 50% decrease in the rate of acute chest syndrome (ACS) complicating vaso-occlusive crisis (VOC) in a tertiary care centre setting like ours.

Another interesting finding in this study is the high discordance rate between the physicians' willingness to recommend prenatal diagnosis and mothers' willingness to accept it. This could be due to the discrepancy between “power to cure or help” by the physicians and “power to care” by the mothers as earlier described by Wonkam and Hurst [9]. This phenomenon is said to occur because the belief by health care providers in their ability to cater for patients with SCD is higher than the belief by the mothers that the care given by medical personnel will be adequate to take care of their wards with SCD [9]. This postulation may not be completely false given the observation that more physicians believed the procedure is safe compared to the mothers. Another reason may be the high financial burden and catastrophic expenditure incurred on SCD among some households as earlier reported [29]. In addition, some of the mothers in this study have to cater for more than one child with SCD.

Furthermore, the various reasons given by the respondents for not practicing prenatal diagnosis could stem from their cultural/religious beliefs. Most Nigerians belong to various religious/cultural groups that hold strict beliefs about the sanctity of life. Unfortunately, we did not examine the religious inclinations of our study participants due to the sensitive nature of such matter in Nigeria at the moment. Nevertheless, as shown in this study, studies from other parts of the world have also hinted on the great impacts some multicultural factors could exert on reproductive choices of women [30, 31]. Furthermore, a handful of the physicians objected the prenatal diagnosis because of ethical concerns and surprisingly also because of religious beliefs, further attesting the complex nature of reproductive choices of women and parents in the study location. These fears may not be far-fetched, given that abortion/termination of pregnancy, for reasons other than to preserve the life of mothers, is still prohibited in Nigeria [32]. Surprisingly, this observation tallies with findings from some developed countries [33] and further raises the need for enforcement of the fundamental reproductive and human rights of women as stipulated by the WHO [34]. Another perspective to the poor performance of prenatal diagnosis in this study could also be the opposition to eugenics by some physicians. Eugenics is defined as policies and practices designed to promote the reproduction of human beings with desirable attributes while avoiding humans with certain undesirable attributes, e.g., SCD and disability, which some people including physicians have campaigned against rigorously because they perceive it as gross infringement on the fundamental right to life of the would-be affected SCD children whose life is terminated through the prenatal technologies/practices [35].

This study is limited by its being questionnaire based with the possibility of recall bias and the respondents not volunteering the whole information. In addition, the study findings cannot be generalized given its limited size and coverage. However, the strength of the study lies in the involvement of women whose studies have shown that they are more reliable at making informed decision about SCD screening and reproductive choices compared to men who are usually under complex pressures at taking such decisions [36, 37]. Despite these limitations, the study established some important dynamics affecting the perception and practice of early SCD in the study locality. In addition, several possibilities for the findings as well as suggestions on some approaches towards addressing the situation were highlighted.

In conclusion, this study shows that the practice of early diagnosis of SCD is still limited in the study locality and among the study participants too. There is need to scale-up early SCD diagnosis in the locality and the country at large by removing legal/constitutional, operational, or policy barriers that could infringe upon its effective implementation.

## Figures and Tables

**Table 1:** Sociodemographic characteristics, practice of early diagnosis, and awareness of the national treatment guidelines for sickle cell disease among physicians

Characteristics of respondents *N* = 135	Frequencies	Percentages (%)
Sociodemographic characteristics		
Designation		
Resident doctors	67	49.6
Medical officers	48	35.6
Consultants	20	14.8
Practice location		
Government hospital	120	88.9
Private hospital	15	11.1
Years of practice		
≤10	73	54.1
11–20	41	30.4
>20	21	15.6
SCD genetic counseling and testing practice		
Routinely recommend it to eligible patients	129	95.6
Occasionally recommend it to patients	6	4.4
Awareness about national newborn screening programme		
Aware	89	65.9
Not aware	46	34.1
Willingness to recommend newborn screening to patients		
Willing	75	55.6
Unwilling	14	10.4
Not sure/unknown	46	34.0
Awareness about national guideline for SCD management		
Aware	96	71.1
Not aware	39	28.9
Awareness about prenatal SCD diagnosis		
Aware	132	97.8
Not aware	3	2.2
Willingness to recommend prenatal SCD diagnosis		
Willing	51	37.8
Unwilling	84	62.2

Notes: SCD = sickle cell disease.

**Table 2:** Mothers' demographic characteristics, awareness, and perception of early SCD diagnosis.

Characteristics of respondents *N* = 164	Frequencies	Percentages (%)
Sociodemographic characteristics		
Level of education		
Tertiary	108	65.9
Secondary	46	28.0
Primary	10	6.1
Marital status		
Married	146	89.1
Single	15	9.1
Divorced/separated	3	1.8
Number of children with SCD		
One	159	97.0
Two	4	2.4
Three	1	0.6
Greater than three	0	0.0
Experience with SCD genetic counseling and testing		
Had participated in it before	154	93.9
Never participated in it before	10	6.1
Awareness of national newborn screening program		
Aware	47	28.7
Not aware	117	71.3
Willingness to offer newborn screening to future children		
Willing	158	96.3
Unwilling	6	3.7
Awareness about government guideline for SCD management		
Aware	23	14.0
Not aware	141	86.0
Awareness about prenatal SCD diagnosis		
Aware	42	25.6
Not aware	122	74.4
Willingness to partake in prenatal SCD diagnosis in the future		
Willing	28	17.1
Unwilling	92	56.1
Undecided/unknown	44	26.8

Notes: SCD = sickle cell disease.

**Table 3:** Perceptions and practice of early diagnosis of sickle cell disease by participants.

Parameters	Physicians *N* = 135 *n* (%)	Mothers *N* = 164 *n* (%)	*p* value
Awareness of national newborn screening program			
Aware	89 (65.9)	47 (28.7)	**<0.0001** ^a^
Not aware	46 (34.1)	117 (71.3)	

Physicians willingness to recommend newborn screening and mothers willingness to partake in it			
Willing	75 (55.6)	158 (96.3)	**<0.0001** ^b^
Unwilling	14 (10.4)	6 (3.7)	
Undecided	46 (34.0))	0 (0)	

Awareness of national guideline for sickle disease management			
Aware	96 (71.1)	23 (14.1)	**<0.0001** ^a^
Not aware	39 (28.9)	141 (85.9)	

Awareness of prenatal sickle cell disease diagnosis			
Aware	132 (97.8)	42 (25.6)	**<0.0001** ^a^
Not aware	3 (2.2)	122 (74.4)	

Physicians willingness to recommend prenatal sickle cell disease diagnosis and mothers willingness to partake in it			
Willing	51 (37.8)	28 (17.1)	**<0.0001** ^b^
Unwilling	84 (62.2)	92 (56.1)	
Undecided	0 (0)	44 (26.8)	

Notes: a = Fisher's exact test, b = chi-square test, and *p* values in the bold font signify statistical significance.

**Table 4:** Reasons given by participants for not practicing prenatal diagnosis of sickle cell disease.

Reasons	Physicians *N* = 84 *n* (%)	Mothers *N* = 92 *n* (%)	*p* value
High cost of procedure	50 (59.5)	63 (68.5)	0.27^a^
Fear of injury to the baby or mother	11 (13.1)	31 (33.7)	**0.0014** ^a^
Negative previous experience	13 (15.5)	2 (2.2)	**0.002** ^a^
Religious beliefs	4 (4.7)	17 (15.0)	**0.0052** ^a^
No reason given	0 (0)	1 (1.1)	1.000^a^
Ethical concerns	21 (25.0)	NA	NA

Notes: some respondents have multiple responses, a = Fisher's exact test, NA = not applicable, and *p* values in the bold font signify statistical significance.

## Data Availability

The data used to support the findings of this study are available from the corresponding author upon request.
